# Four Directions, One Solution: Enabling Rapid Diffusion Tensor MRI for Ultra‐Low Field Using Deep Learning

**DOI:** 10.1002/mrm.70417

**Published:** 2026-05-10

**Authors:** Joshua Mawuli Ametepe, James Gholam, Leandro Beltrachini, Mara Cercignani, Derek Kenton Jones

**Affiliations:** ^1^ Cardiff University Brain Research Imaging Centre, School of Psychology Cardiff University Cubric Cardiff UK; ^2^ School of Physics and Astronomy Cardiff University Cardiff UK

## Abstract

**Purpose:**

This study revisits the tetrahedral encoding strategy originally proposed to accelerate Diffusion Tensor Magnetic Resonance Imaging (DT‐MRI) by reducing the requisite number of diffusion‐weighted measurements to four. We examine its practical limitations and explore how artificial intelligence (AI) can extend its utility. Specifically, we employ deep learning (DL) to estimate diffusion tensor parameters from four tetrahedrally arranged measurements rather than the conventional six or more, enabling substantially shorter scan durations. This approach is particularly relevant for low‐field (LF) and ultra‐low‐field (ULF) MRI, where long acquisitions are needed to offset low SNR, and for non‐compliant populations where extended scan times are impractical.

**Methods:**

To overcome the numerical instabilities of traditional tetrahedral encoding, we developed DL models to predict axial and radial diffusivities and the principal eigenvector from four measurements. Synthetic training data spanned a wide range of diffusion tensors with uniformly distributed eigenvalues and orientations. Models were evaluated on digital phantoms and in vivo datasets acquired at 3T and 64 mT.

**Results:**

The DL‐based approach improved accuracy in estimating diffusivities, fractional anisotropy, and orientation compared to conventional tetrahedral methods, particularly under low‐SNR conditions. Residual errors persisted when the principal eigenvector aligned with scanner axes, reflecting inherent geometric constraints.

**Conclusion:**

By revisiting and refining tetrahedral encoding through AI‐driven strategies, this work demonstrates the feasibility of rapid DT‐MRI using only four directions. These findings highlight opportunities for clinically viable diffusion imaging in time‐constrained or resource‐limited settings, while identifying key limitations for future research.

## 1 Introduction

Diffusion‐weighted imaging (DWI) is sensitive to the microscopic motion of water molecules within tissue, thus providing a unique contrast which complements T_1_ and T_2_‐weighted MRI [1]. As diffusion is anisotropic in brain white matter [2], typically three images with orthogonal diffusion directions are acquired and averaged to provide rotationally‐invariant mean apparent diffusion coefficient (ADC) maps [3]. Clinically, DWI has become the standard diagnostic scan for acute stroke and is increasingly acquired in other conditions such as multiple sclerosis, brain tumors, and dementia [4].

Going beyond scalar ADC maps, diffusion tensor MRI (DT‐MRI) [5], provides richer information by estimating the full diffusion tensor, enabling quantification of diffusion anisotropy (e.g., fractional anisotropy) and the inference of tissue orientation [6]. However, estimation of the full diffusion tensor requires a minimum of 6 diffusion‐weighted images [5]. On higher field systems, with accompanying higher signal to noise ratio (SNR), and therefore less need to collect multiple averages of a signal, this requirement can be met within practical acquisition times. However, conducting DT‐MRI at low fields/ultra‐low fields (LF/ULF) presents significant challenges. DWI encodes the effects of diffusion as a signal attenuation, and therefore inherently results in low SNR images. This SNR problem is further exacerbated at low/ultra‐low field [7]. While increasing the number of averages can offset low SNR, this becomes an issue in non‐compliant populations, like infants, children, or individuals who find it difficult to stay still for long periods.

One potential solution is to reduce the number of diffusion‐weighted directions acquired. In this context, this study re‐visits the tetrahedral encoding approach originally proposed by Conturo et al. [8]. This method hinges on the assumption of cylindrical symmetry of the diffusion tensor, allowing for a reduction in the minimum number of required measurements from six to four [8], effectively enabling the estimation of anisotropy and tissue orientation with just one extra measurement compared to the standard clinical ADC protocol. However, while tetrahedral encoding is appealing in theory, practical limitations remain. Prior work [9] has shown that this method can lead to errors in anisotropy (FA) estimates, but did not fully explore the bias in tensor orientations, nor the origin or mitigation of such errors.

Here we provide an explanation for this bias. Next, using Monte Carlo simulations, we compare the noise sensitivity of the reduced tetrahedral encoding approach to that of a full six‐direction encoding technique. To overcome these limitations and improve robustness in low‐SNR environments, we then leverage deep learning (DL). Using synthetically generated diffusion data encompassing a wide range of tensor shapes and orientations, we train DL models to estimate diffusivities and principal eigenvectors from only four diffusion‐weighted images. We validate this approach using both digital phantoms and in vivo data acquired at 3T and 64 mT. This hybrid strategy, combining physical modeling and data‐driven inference, offers a path toward rapid, reliable DT‐MRI, particularly in LF/ULF settings and in populations for whom traditional acquisition schemes are impractical.

## 2 Theory

Estimating a general diffusion tensor requires at least six diffusion‐weighted images and one b = 0 s/mm^2^ image for accurate estimation [5, 10]. However, in 1996, tetrahedral encoding for DT‐MRI was introduced, allowing for tensor estimation from just four diffusion‐weighted images under the assumption of cylindrical symmetry of the tensor. Under this assumption, the secondary and tertiary eigenvalues are equal (i.e., λ_2_ = λ_3_), and as such, the diffusion tensor can be reduced from six to four independent variables [8]: The principal eigenvalue (λ_1_), the radial eigenvalue (λ_2_), and the two orientation angles of the principal eigenvector (θ and ∅). This reduction allows for tensor estimation with fewer measurements, provided that the diffusion gradient orientations are isotropically distributed. This condition is satisfied by a tetrahedral gradient configuration, in which the four directions correspond to the vertices of a regular tetrahedron.

In this configuration, for each acquisition, all three gradients (*x*, *y*, *z*) are applied simultaneously at the same amplitude, producing four unit vectors:

(1) $$g_{1}=c\;\left(\begin{array}{l} 1\\ 1\\ 1 \end{array}\right),g_{2}=c\left(\begin{array}{l} -\;1\\ -\;1\\ 1 \end{array}\right),g_{3}=c\;\left(\begin{array}{l} 1\\ -\;1\\ -\;1 \end{array}\right),g_{4}=c\left(\begin{array}{l} -\;1\\ 1\\ -\;1 \end{array}\right),$$

where $c=1/\sqrt{3}$.

In this case, we can relate the ADC along each encoding axis with the DT elements, leading to

(2a) $$D_{1}=\frac{1}{3}\left[\left(D_{\mathrm{xx}}+D_{\mathrm{yy}}+D_{\mathrm{zz}}\right)+2\left(D_{\mathrm{xy}}+D_{\mathrm{xz}}+D_{\mathrm{yz}}\right)\right],$$

(2b) $$D_{2}=\frac{1}{3}\left[\left(D_{\mathrm{xx}}+D_{\mathrm{yy}}+D_{\mathrm{zz}}\right)+2\left(D_{\mathrm{xy}}-D_{\mathrm{xz}}-D_{\mathrm{yz}}\right)\right],$$

(2c) $$D_{3}=\frac{1}{3}\left[\left(D_{\mathrm{xx}}+D_{\mathrm{yy}}+D_{\mathrm{zz}}\right)+2\left(-D_{\mathrm{xy}}-D_{\mathrm{xz}}+D_{\mathrm{yz}}\right)\right],$$

(2d) $$D_{4}=\frac{1}{3}\left[\left(D_{\mathrm{xx}}+D_{\mathrm{yy}}+D_{\mathrm{zz}}\right)+2\left(-D_{\mathrm{xy}}+D_{\mathrm{xz}}-D_{\mathrm{yz}}\right)\right].$$

It can be easily seen that the arithmetic mean of these four expressions is the mean diffusivity [8].

$\mathrm{MD}=\frac{1}{3}\left(D_{\mathrm{xx}}+D_{\mathrm{yy}}+D_{\mathrm{zz}}\right)$, which is also the first term in each of the equations. Once this first term is estimated, it is trivial to estimate the off‐diagonal elements through manipulation of Equations ((2a), (2b), (2c), (2d)).

Under the assumption of cylindrical symmetry, the principal eigenvector and parallel and radial diffusivities can be accurately estimated from the off‐diagonal components using the following expressions as shown by Conturo et al. [8].

(3a) $$\emptyset =\tan^{-1}\frac{D_{\mathrm{xy}}\sqrt{D_{\mathrm{xz}}^{2}+D_{\mathrm{yz}}^{2}}}{D_{\mathrm{xz}}D_{\mathrm{yz}}},$$

(3b) $$\theta =\tan^{-1}\frac{D_{\mathrm{yz}}}{D_{\mathrm{xz}}},$$

(3c) $$\lambda_{∥}=\mathrm{MD}+2\eta ,$$

(3d) $$\lambda_{⊥}=\mathrm{MD}-\eta ,$$

where

(3e) $$\eta =\frac{1}{3}\left(\frac{D_{\mathrm{xy}}D_{\mathrm{xz}}}{D_{\mathrm{yz}}}+\frac{D_{\mathrm{xy}}D_{\mathrm{yz}}}{D_{\mathrm{xz}}}+\frac{D_{\mathrm{xz}}D_{\mathrm{yz}}}{D_{\mathrm{xy}}}\right).$$

### 2.1 The Axis Problem

A problem is encountered when a tensor's eigenvectors are oriented along the **x**, **y**, and **z** axes since the tensor's off‐diagonal components (the denominator in Equations 3a, 3b, and 3e become zero). The closer the off‐diagonal elements are to zero, the larger the error [9]. Moreover, the standard tetrahedral approach hinges on the differential signal attenuations across different gradient directions. Looking at Equations ((2a), (2b), (2c), (2d)), when the tensor is rotated such that all off‐diagonal elements are zero, each ADC is equal to the mean diffusivity, incorrectly suggesting isotropy.

**FIGURE 1:**
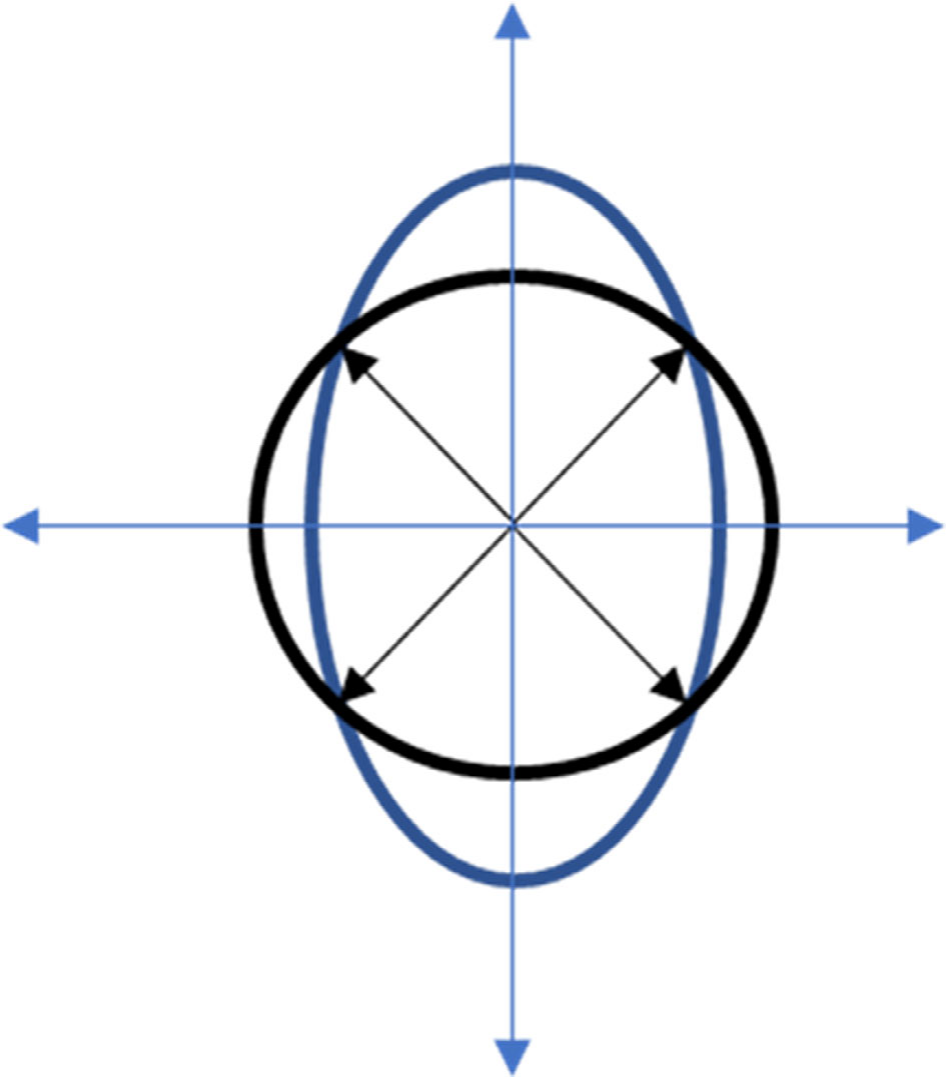
A 2D visual representation of the axis problem. The blue curve shows an ellipse aligned with the vertical axes, with gradient vectors at 45° angles extending from the center to the edges. The black curve is a circle that we can fit to the same gradient vectors. This illustrates that with the tetrahedral gradient configuration, when the tensor aligns with the scanner's axes, the measurements correspond to those of a sphere, demonstrating the isotropic appearance despite anisotropy in the actual data.

Even when only the principal eigenvector aligns with the scanner's axes, the same issue persists. Under ideal circumstances, variations in the second and third eigenvalues would lead to differences in the measured signals. However, assuming cylindrical symmetry equates the second and third eigenvalues, rendering the orientations of the second and third eigenvectors inconsequential (Figure 1). Therefore, when the tensor's principal eigenvector aligns closely with any of the scanner's axes, measurement variance decreases, resulting in a more isotropic appearance. This is a mathematical limitation of using only four measurements in DT‐MRI.

This relationship is only valid if the sampling vectors are perfectly isotropically distributed in the measurement space of the tissue. If the participant moves during scanning, with an element of rotation in that movement, the relative orientation of the tissue to the gradient vectors violates this isotropic condition. This is a significant limitation, particularly in real‐world scenarios where participant movement is common.

## 3 Methods

### 3.1 Deep Learning

Deep learning methods are increasingly being proposed as powerful alternatives for fitting diffusion MRI (dMRI) models to data, due to their ability to capture complex, non‐linear relationships between imaging signals and tissue microstructure. Traditional model‐fitting approaches, such as non‐linear least squares, can be computationally intensive and sensitive to noise, especially when dealing with complex models or limited data [11, 12]. In contrast, deep neural networks can be trained on large datasets, either simulated or experimental, to learn direct mappings from diffusion‐weighted signals to model parameters. Once trained, these models offer rapid inference, robustness to noise, and potential generalizability across different acquisition schemes, making them attractive tools for accelerating and improving the accuracy of microstructural imaging in both research and clinical settings.

We developed a DL model to predict parallel and radial diffusivities (λ_1_ and λ_2_), as well as the principal eigenvector, using four diffusion‐weighted images. The universal approximation theorem stipulates that we can find an approximation to a function using neural networks [13]. Our goal was to leverage a neural network to estimate a better approximation of the diffusivities and the principal eigenvector, reducing the errors observed in the standard tetrahedral approach. This required a representative dataset for training, which would typically be collected from a variety of brain samples. However, biases from data collection methods, scanner settings, noise, and other image artifacts can affect accuracy. To mitigate against these biases, we used synthetic data for model training.

The first step involved generating tensors with uniformly distributed eigenvalues and eigenvector orientations. The three eigenvectors were uniformly dispersed in three‐dimensional space and eigenvalues were sampled within a predefined range for each tensor. Our training dataset comprised 14 million diffusion tensors. For each tensor, the angular parameters θ, ∅, and Ψ were uniformly sampled between 0° and 360°. Eigenvalues were sampled within a range of 1.0 × 10^−5^ mm^2^/s to 3.0 × 10^−3^ mm^2^/s to avoid biases while covering expected voxel values [14]. The sampled values were ordered as the first, second, and third eigenvalues, with the mean of the second and third eigenvalues used to simulate a cylindrically symmetric tensor. Tetrahedral gradient vectors (see Equation (1)) were used to generate our diffusion‐weighted signal values.

To simulate the impact of participant motion (i.e., rotation of the participant's reference frame relative to the isotropically‐distributed sampling directions), a randomly generated rotation matrix was applied to the four gradient vectors in each dataset. These matrices were generated by specifying rotation angles between 0° and 20° in the *x*, *y*, and *z* directions.

Using a b‐value range of 500 s/mm^2^ to 1000 s/mm^2^, and a predetermined b = 0 s/mm^2^ signal value, for each simulated tensor, we derived the corresponding four diffusion‐weighted signals. To simulate realistic conditions, Rician noise was added, based on a chosen SNR of 5 (typical for an image collected on a 64 mT system [7]).

### 3.2 Feature Engineering

Organizing features effectively is crucial for our DL algorithm's performance. Our input feature set includes a b = 0 s/mm^2^ signal value, four diffusion‐weighted imaging (DWI) signal values, a b‐value, and four gradient vectors (each with *x*, *y*, *z* components), totaling 18 distinct features per sample, each with its own unique range.

To promote model convergence and ensure fair consideration of all features, we adopted a standardized approach. We converted all inputs into vectors, framing the problem geometrically. These four vectors represent gradient directions, with their magnitudes indicating the ADC. In our dataset, each of these vectors (**v**_**1**_, **v**_**2**_, **v**_**3**_, **v**_**4**_) corresponds to a gradient vector, where the vector length represents the ADC along its respective axis (D_i_). So, for each gradient direction (**g**_**i**_), the input vector is

$$v_{i}=D_{i}g_{i},$$

For i = 1,…,4.

This approach streamlines our input features to 12 (four vectors each with *x*, *y*, *z* components), ensuring uniformity across our dataset. By reducing feature count, we enhance model convergence and ensure that each feature contributes equally to the output. This also means that the model is not directly dependent on scanner field strength since the inputs are based on normalized ADC values and gradient vectors. We developed a multi‐layer perceptron trained on these standardized input data, creating two distinct models: one for predicting diffusivities, and another for estimating the principal eigenvector. The diffusivity model did not enforce the assumption of symmetry on the dataset, whereas it was enforced for the principal eigenvector model.

Training separate models allowed us to customize loss functions tailored specifically to diffusivities or vectors. Versions of both models were trained on the noisy and noise‐free versions of the data. For diffusivities, the loss function was defined as follows:

(4) $$L=\frac{1}{N}\sum_{i=1}^{N}\left[\frac{1}{\alpha }\left(\frac{1}{3}\left\|\lambda_{i}-\hat{\lambda_{i}}\right\|\right)+\left|\mathrm{FA}\left(\lambda_{i}\right)-\mathrm{FA}\left(\hat{\lambda_{i}}\right)\right|\right],$$

where $\lambda_{i}$ are the predicted eigenvalues, $\hat{\lambda_{i}}$ are the target eigenvalues, FA is the estimated fractional anisotropy from the eigenvalues, and α is the upper‐bound of the eigenvalues used in the training data, which is 3.0 × 10^−3^ mm^2^/s in this case. This was used so that the eigenvalue part of the loss will be in a similar numerical range to the “FA” part of the loss, so that neither part will dominate the training.

This loss enforces accuracy in both the predicted eigenvalues and the fractional anisotropy estimated from them.

Conversely, predicting the principal eigenvector presents unique challenges due to its interrelated components and the antipodal symmetry of the tensor [15, 16]. Training on the two polar angles of the vector could result in two sets of θ and ∅, both viable solutions, potentially impeding model convergence. To address this, Cartesian coordinates of the tensor were used as the output of the model and the cost function was a cosine distance metric defined as:

(5) c=1−|a.b|,

where **a** is the true vector, **b** is the vector predicted by the model, and the symbol “·” represents the dot product. Since the loss function only focuses on direction, the model's output must be normalized into a unit vector.

TensorFlow [17] was used to construct and train these models, leveraging its powerful capabilities in handling such neural network architectures and computations. The model structure is shown in the Supporting Information section. The learning rate was 0.001 and training was conducted with the Adam optimizer [18]. Each model was trained for 16 epochs.

### 3.3 Tests

#### 3.3.1 Noise Susceptibility—Six Directions Versus Four Directions

We conducted a Monte Carlo simulation to compare differences in the effects of noise on diffusivity estimation when using 6 and 4 diffusion‐encoding directions. Beginning with a diffusion tensor with specified fractional anisotropy and eigenvalues, we set the trace of the tensor to 0.0021 mm^2^/s [19, 20]. Cylindrical symmetry was enforced, and we employed gradient vectors oriented along cube edges [21], a b‐value of 1000 s/mm^2^, and an arbitrary b = 0 s/mm^2^ signal amplitude. We generated six and four diffusion‐weighted signals using the formula:

(6) $$S=S_{0}e^{-b\left(\mathrm{gD}g^{T}\right)},$$

where

(7) $$\mathrm{SNR}=\frac{\mu }{\sigma },$$

with μ representing the mean of the b = 0 s/mm^2^ signal and σ the standard deviation. Given a specific b = 0 s/mm^2^ signal amplitude and a predetermined SNR, we added Rician noise to our signals [19, 20].

For each SNR, the eigenvalues were estimated using ordinary least squares for the six‐direction approach [22]. The standard tetrahedral method published by Conturo et al. [8] was used for the four‐direction approach to estimate parallel and radial diffusivities. The process was repeated for 2000 iterations.

#### 3.3.2 Noise Susceptibility—Standard Tetrahedral Approach Versus Deep Learning Model

Using the same method, we generated four‐directional signals for a diffusion tensor with a fractional anisotropy of 0.75 at varying SNR values. We estimated parallel and radial diffusivities using both the standard tetrahedral approach and our DL model. These tests employed a non‐cylindrically symmetric tensor. The aim of this test was to see how well the model performed in varying SNR conditions, as compared to the standard tetrahedral approach.

#### 3.3.3 Orientation Based Error

Given the errors in estimates of the tensor elements when tensors are oriented close to the scanner's axes in the standard tetrahedral method, we tested whether our DL model reduces these errors. We generated non‐cylindrically symmetric diffusion tensors with varying fractional anisotropy (FA) values and rotated each tensor by varying the polar angles (θ and ∅) of the principal eigenvector. Four‐directional signals were generated for each orientation, and FA and tensor orientation were predicted using the standard tetrahedral approach and our DL model. We also rotated one of the gradient vectors by 12° to simulate transformations due to head motion, particularly in non‐compliant participants.

#### 3.3.4 Tests on Experimental Image Data

The model was tested on a digital phantom from the Human Connectome Project, containing a DWI dataset with 128 gradient orientations [23] and on images collected at Cardiff University from a single participant (male, 24 year‐old) at 64 mT on a Hyperfine Swoop system, and at 3T on a Siemens Connectom scanner with 300 mT/m gradients. The 64 mT and 3T Connectom study was approved by Cardiff University's School of Psychology ethics committee. After providing written informed consent, the participant was instructed to remain still during the scans.

For the 64 mT scans, data were acquired using a diffusion‐weighted fast spin echo (FSE) sequence [24] with tetrahedral encoding and a b value of 900 s/mm^2^. The DWI sequence employed for this was a 3D multi‐shot diffusion‐weighted, non‐Carr–Purcell–McGill–Bloom (CPMG), self‐navigated fast spin echo, employing a non‐Cartesian k‐space trajectory. A total of 212 shots were used for each volume, using a split echo train with 56 echoes across a TR of 800 ms, spanning 28 unique phase encodings. The effective echo time was 82 ms. The FoV used was 220 × 200 × 180 mm, in AP/SI/RL directions, respectively. Data were reconstructed at a resolution of 3 × 3 × 6 mm. The readout was fully sampled and oriented in the RL direction, whereas the phase encoding directions were oversampled by 330%. Minimal post‐processing was performed, limited to image co‐registration using FSL's FLIRT [25], without applying denoising methods.

For the 3T Connectom scans, data were acquired with a pulsed‐gradient spin echo echo‐planar imaging (PGSE EPI) sequence with a b value of 1000 s/mm^2^. The resolution for the images was 2 × 2 × 2 mm with an echo time of 59 ms. The sampling comprised diffusion‐weighted images obtained using 128 tetrahedral gradient vectors (128 sets of four directions). Of these, 64 were repeated with the same gradient orientations, while for the remaining 64, the gradient vectors were rotated about the origin. This setup allowed us to assess the temporal consistency of the results when maintaining identical gradient orientations and the robustness of the model across different tetrahedral orientations. These images were denoised using the MPPCA algorithm [26] and image co‐registration was performed with FSL's FLIRT [25].

The variance in the estimation of anisotropy was computed for the two conditions. To assess variability in orientation estimates, a mean orientation was computed per voxel using singular value decomposition (SVD), and angular deviations were calculated between each predicted orientation and the mean while accounting for antiparallel symmetry of the vectors. The variation in the estimation of orientation was computed as the average of the angular deviation from the mean orientation.

Another dataset was acquired on the 3T Connectom scanner with a PGSE EPI sequence, a b‐value of 1000 s/mm^2^, and a resolution of 2 × 2 × 2 mm. With this dataset, there were 60 different sets of tetrahedral gradient vectors evenly rotated about the origin, making a total of 240 directions. Using this dataset, a DTI fit was performed on each set of four directions using the model and the standard (Conturo's) tetrahedral approach. We used an ordinary least‐squares estimate on the full 240 direction dataset as our “ground truth”. For each method, we computed a mean eigenvector from the estimates of the principal tensor orientation. We then computed angular deviations for each voxel and took the 95th percentile of these deviations for each method. This allowed us to plot a cone of uncertainty [27] for each voxel for each method. A comparison of the accuracy in estimation of anisotropy, principal tensor orientation and the cone of uncertainty is shown in Figure 10.

## 4 Results

This section shows the results of the various tests that were carried out.

### 4.1 Noise Susceptibility—Six Directions Versus Four Directions

**FIGURE 2:**
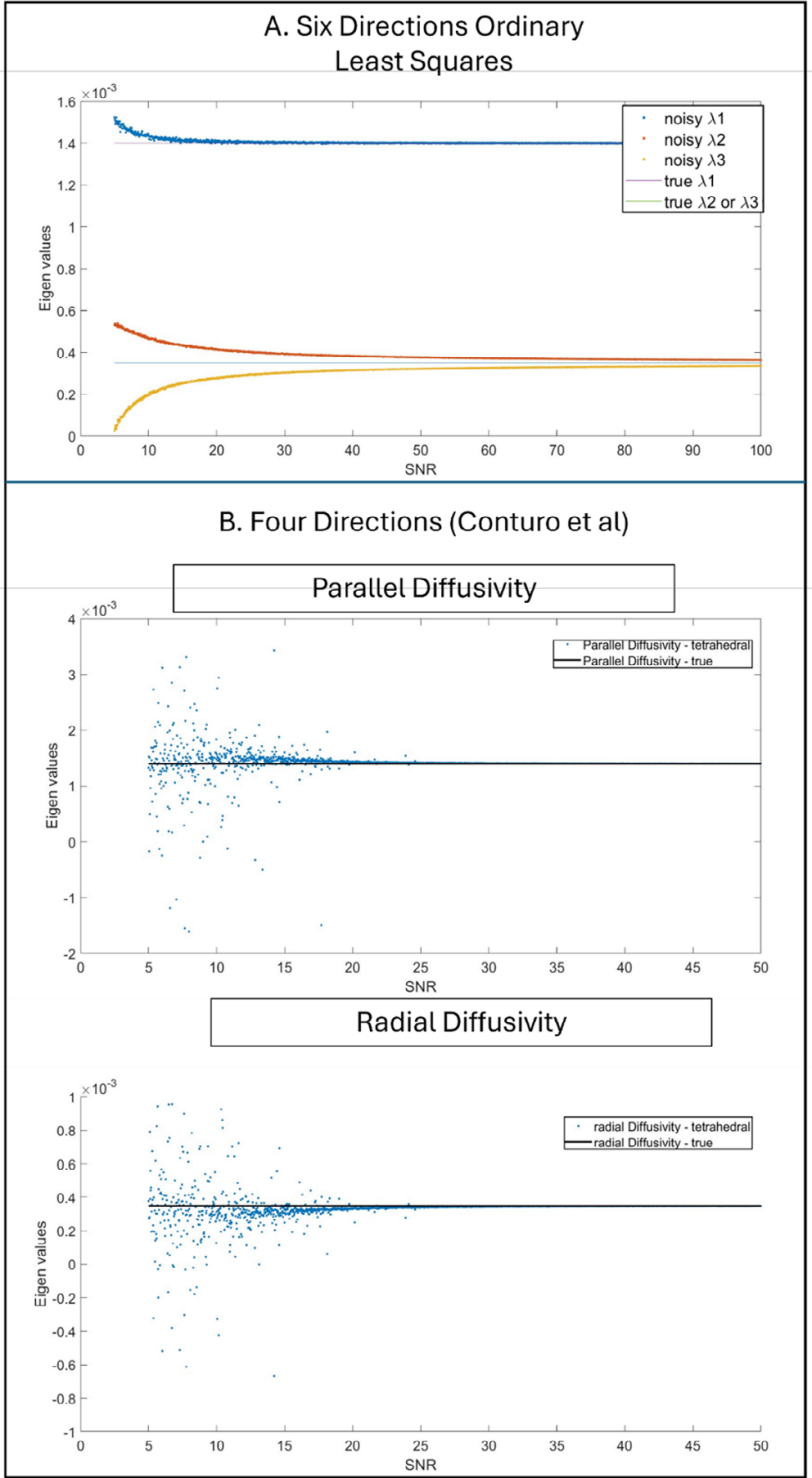
(A) Effect of noise on the eigenvalue estimations (units = mm^2^/s) on a cylindrically symmetric tensor after a linear least squares estimation with six diffusion‐weighted images. FA = 0.71, Trace = 0.0021 mm^2^/s. (B) Effect of noise on the estimations of parallel and radial diffusivities (units = mm^2^/s) using the Conturo et al. tetrahedral method on a cylindrically symmetric tensor. FA = 0.71, Trace = 0.0021 mm^2^/s. The graph is separated into two for better clarity and comparison.

Figure 2A shows results from the six‐direction linear least squares estimation, while Figure 2B shows the four‐direction estimation using the standard tetrahedral approach. Due to significant differences in magnitudes of the eigenvalues, the parallel and radial diffusivities for the four‐direction estimates were plotted separately. The four‐direction estimates exhibit a greater deviation from true values at low SNR compared to the six‐direction estimates, demonstrating the enhanced sensitivity of the standard tetrahedral approach to low SNR and its limitations in low‐field datasets.

### 4.2 Noise Susceptibility—Standard Tetrahedral Approach Versus Deep Learning Model

**FIGURE 3:**
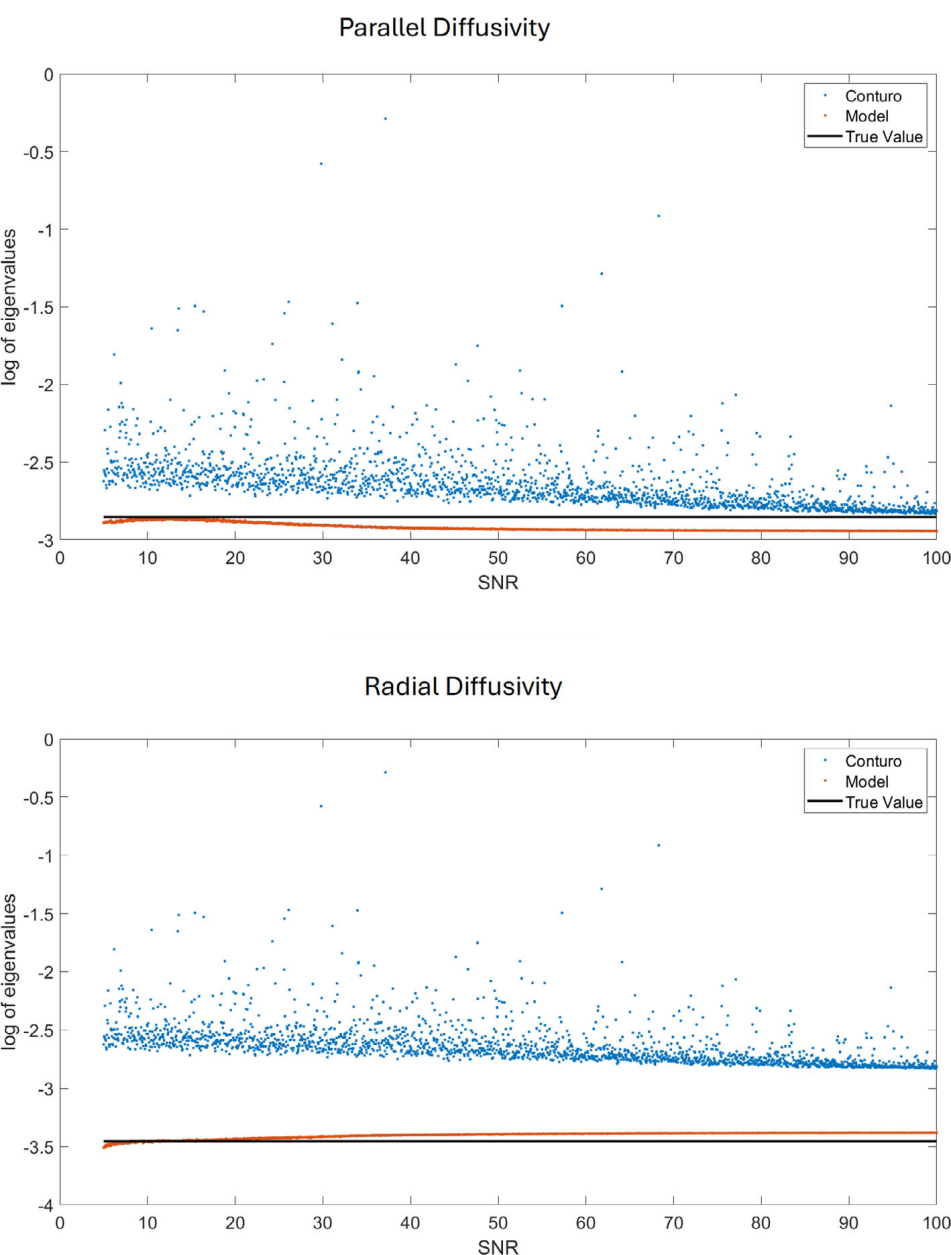
Monte Carlo comparison of the estimated parallel and radial diffusivities (units = mm^2^/s) of the DL model and the Conturo et al. tetrahedral approach, with the true value of FA = 0.75, Trace = 0.0021 mm^2^/s. The DL model shows a greater robustness to noise as compared to the standard tetrahedral approach.

Figure 3 compares the predicted parallel and radial diffusivities from both the DL model and the standard tetrahedral approach across various SNR levels. The DL model shows greater stability and less deviation from the ground truth at low SNR compared to the standard tetrahedral approach, indicating better performance in noisy conditions.

### 4.3 Orientation Based Error

**FIGURE 4:**
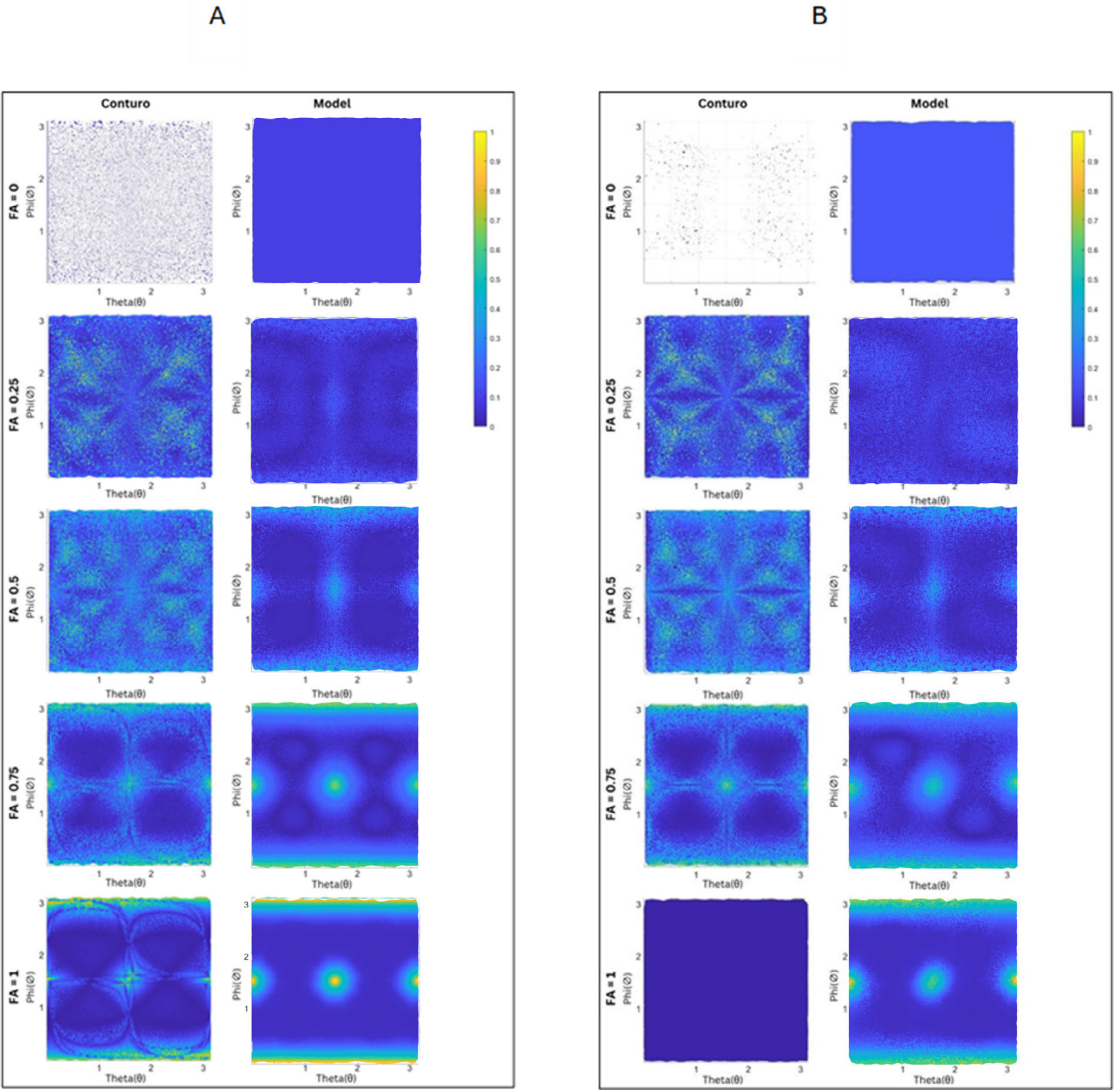
The absolute value of the FA error for various orientations of the principal eigenvector (θ, Φ). Trace = 0.0021 mm^2^/s. In A, the gradient vectors correspond to a perferct tetrahedron. In B, one of the gradient axes was rotated 12° about the *x*‐axis to simulate transformations due to registration.

**FIGURE 5:**
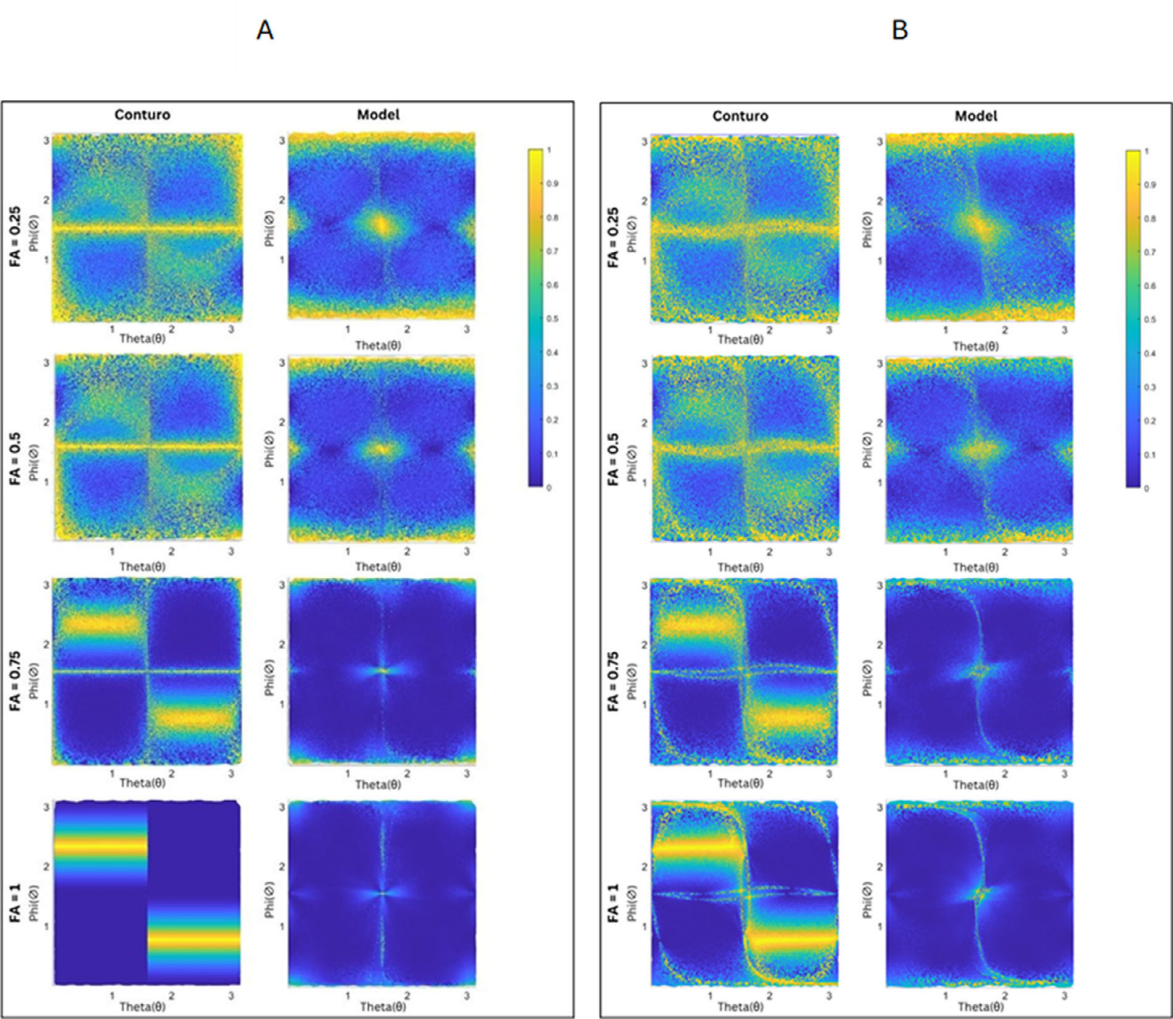
The angular error as the cosine distance between the predicted principal eigenvector and the true vector for various orientations of the vector (θ, Φ). Trace = 0.0021 mm^2^/s. In A, the gradient vectors correspond to a perferct tetrahedron. In B, one of the gradient axes was rotated 12° in the *x*‐axis to simulate transformations due to registration.

Figures 4 and 5 compare the DL and standard tetrahedral approach in predicting fractional anisotropy (FA) and fiber orientations, respectively, for tensors oriented both close to and away from the scanner's axes. Although errors persist, the DL model significantly reduces these errors compared to the standard tetrahedral approach, demonstrating higher accuracy in predictions.

### 4.4 Image Quality

**FIGURE 6:**
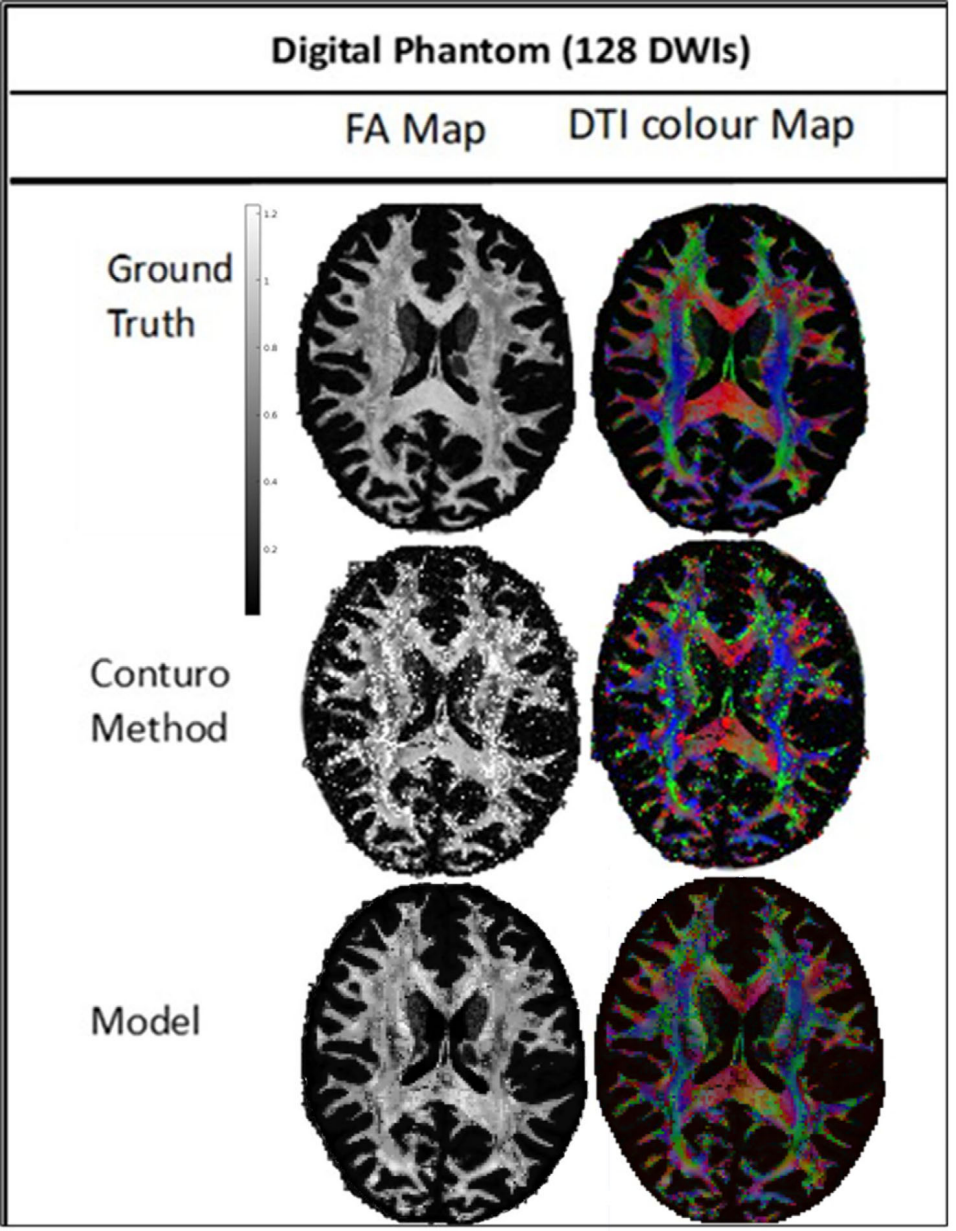
The results of using the DL‐model on the digital phantom as compared with those from the Conturo et al. tetrahedral method.

**FIGURE 7:**
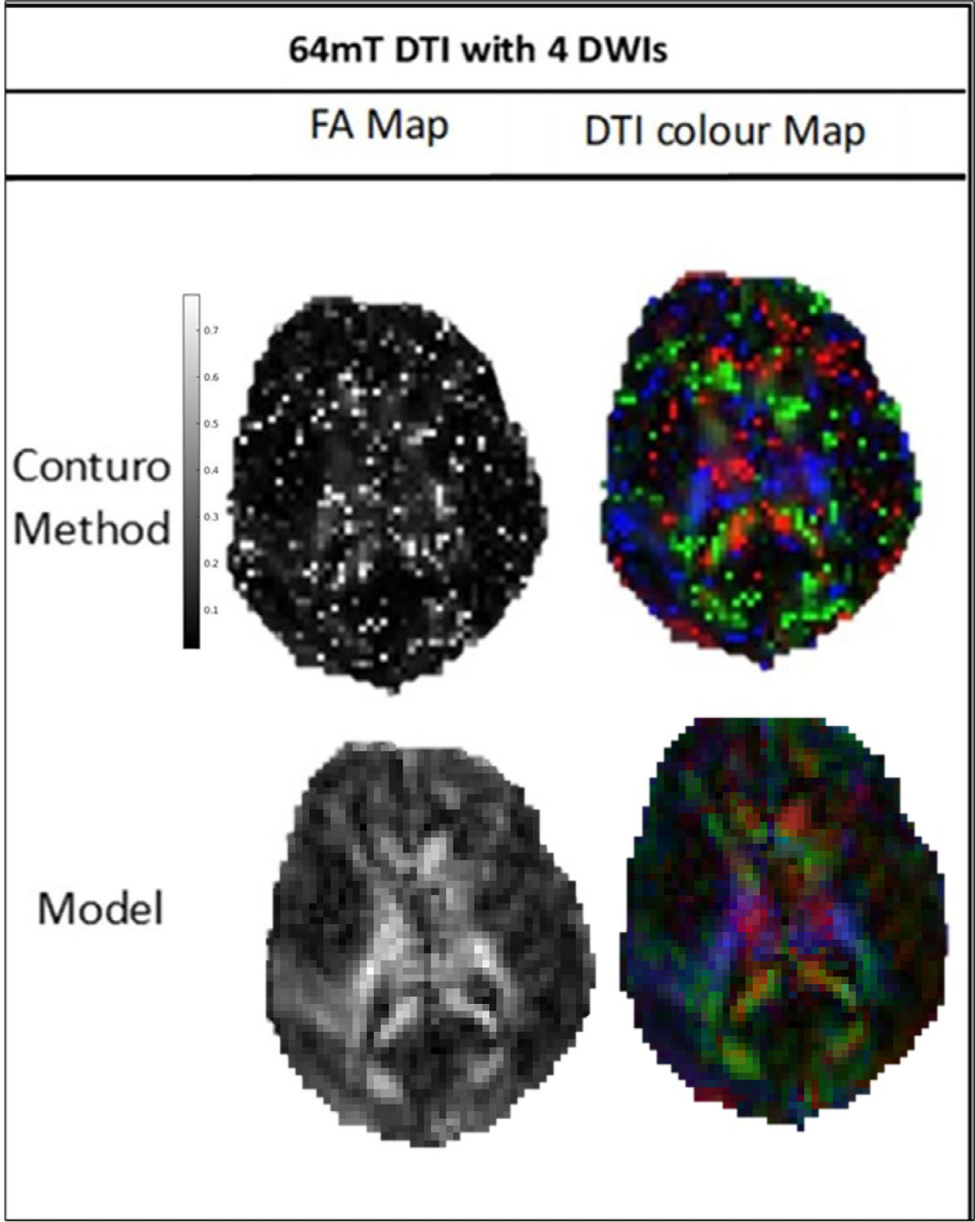
The results of the model at 64 mT compared with the Conturo et al. tetrahedral method.

**FIGURE 8:**
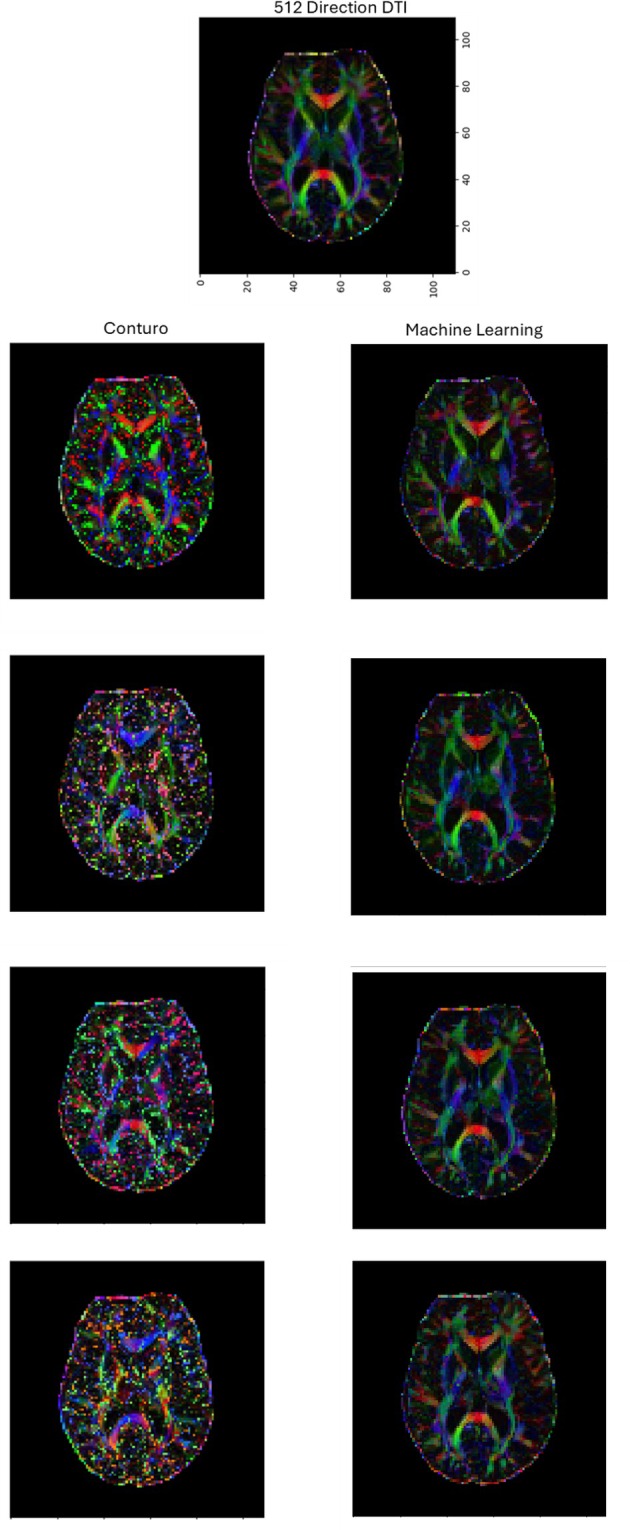
Comparison of the results of the DL model with those of the Conturo et al. method on the 3T images. Each row shows the results for different orientations of the four tetrahedral gradient vectors. The image at the top shows the result after doing an ordinary least squares fit on the full 512‐direction dataset.

Visual inspection of the digital phantom results in Figure 6 shows that the FA and orientation color maps generated by the DL model more closely match the ground truth compared to those produced by the standard tetrahedral approach. In images taken at 64 mT, shown in Figure 7, the standard tetrahedral approach produces what appears to be unstructured noise in FA maps, whereas the DL model highlights distinct anatomical structures, including the genu, splenium, internal capsule, and projections to the occipital cortex, indicating superior performance in accurately representing FA and fiber orientations. Figure 8 shows our tests on 3T images. We see much less noisy anisotropy and orientation maps from the DL model compared to the standard tetrahedral approach. However, we do also see some rotational variance in the results for different orientations of the tetrahedral sampling schemes, which is fully in alignment with the in silico results presented in Figures 4 and 5.

### 4.5 Comparing Measurement Variance Versus Rotational Variance

**FIGURE 9:**
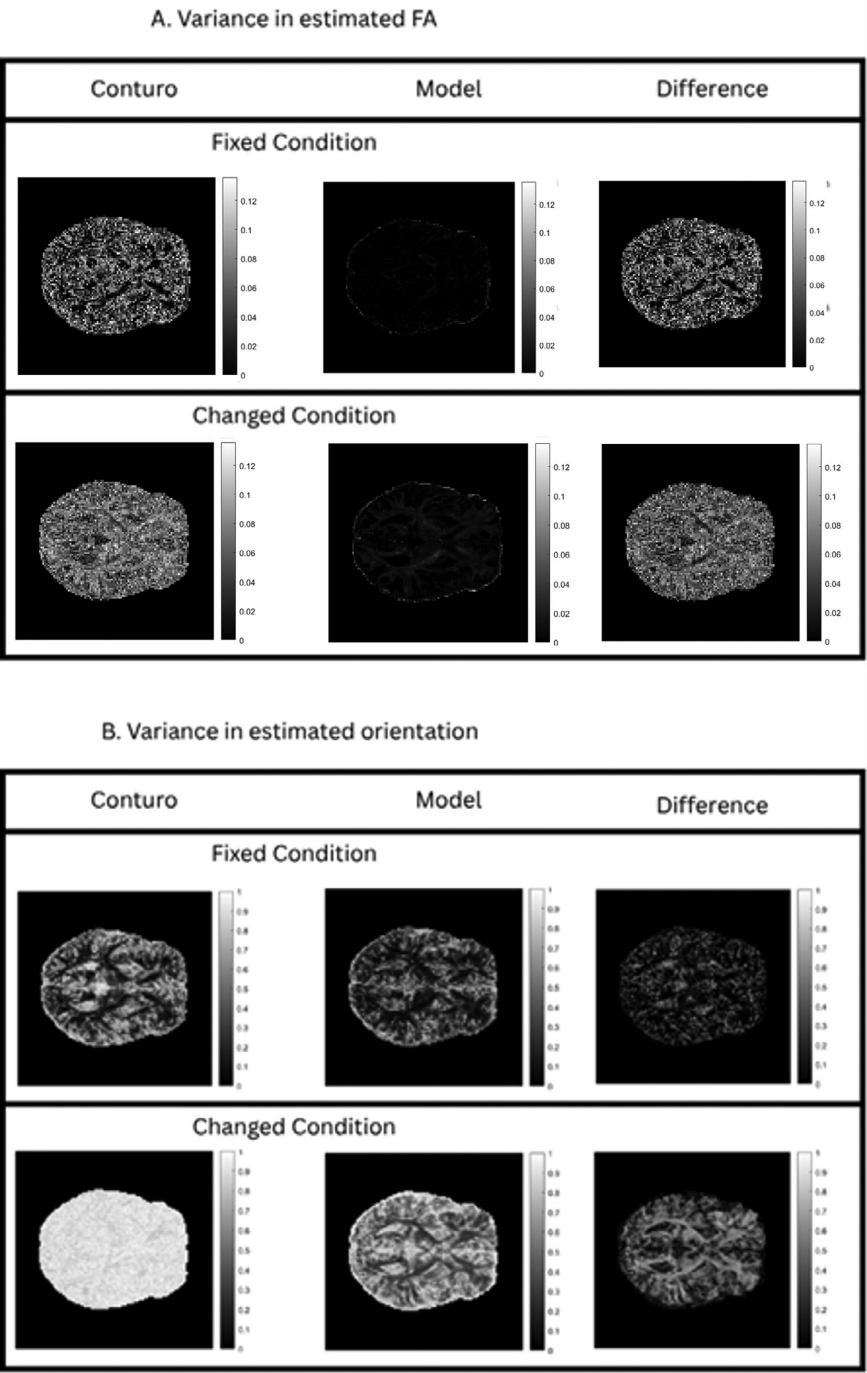
(A) The variance in the estimation of fractional anisotropy for the condition where the same tetrahedral gradient vectors are repeated (referred to in the figure as “fixed condition”), and the condition where the vectors were rotated (referred to as “changed condition”) for both the model and the Conturo method. The Difference Image is also shown (Conturo—Model). (B) The variance in the estimation of orientation for the condition where the same tetrahedral gradient vectors are repeated (referred to in the figure as “fixed condition”), and the condition where the vectors were rotated (referred to as “changed condition”) for both the model and the Conturo method. The Difference Image is also shown (Conturo—Model).

The analysis of the 3T Connectom data collected in Section 3.3.4 is presented in Figure 9.

In Figure 9, it can be seen that for both the estimation of FA and the estimation of orientation, the DL approach displays lower variance than the standard tetrahedral approach both for the conditions where we rotate the gradient vectors and where we repeat the same gradient vectors. This shows improved stability in the results when using the DL approach compared to the standard tetrahedral approach.

### 4.6 In Vivo Test: Error and Uncertainty

**FIGURE 10:**
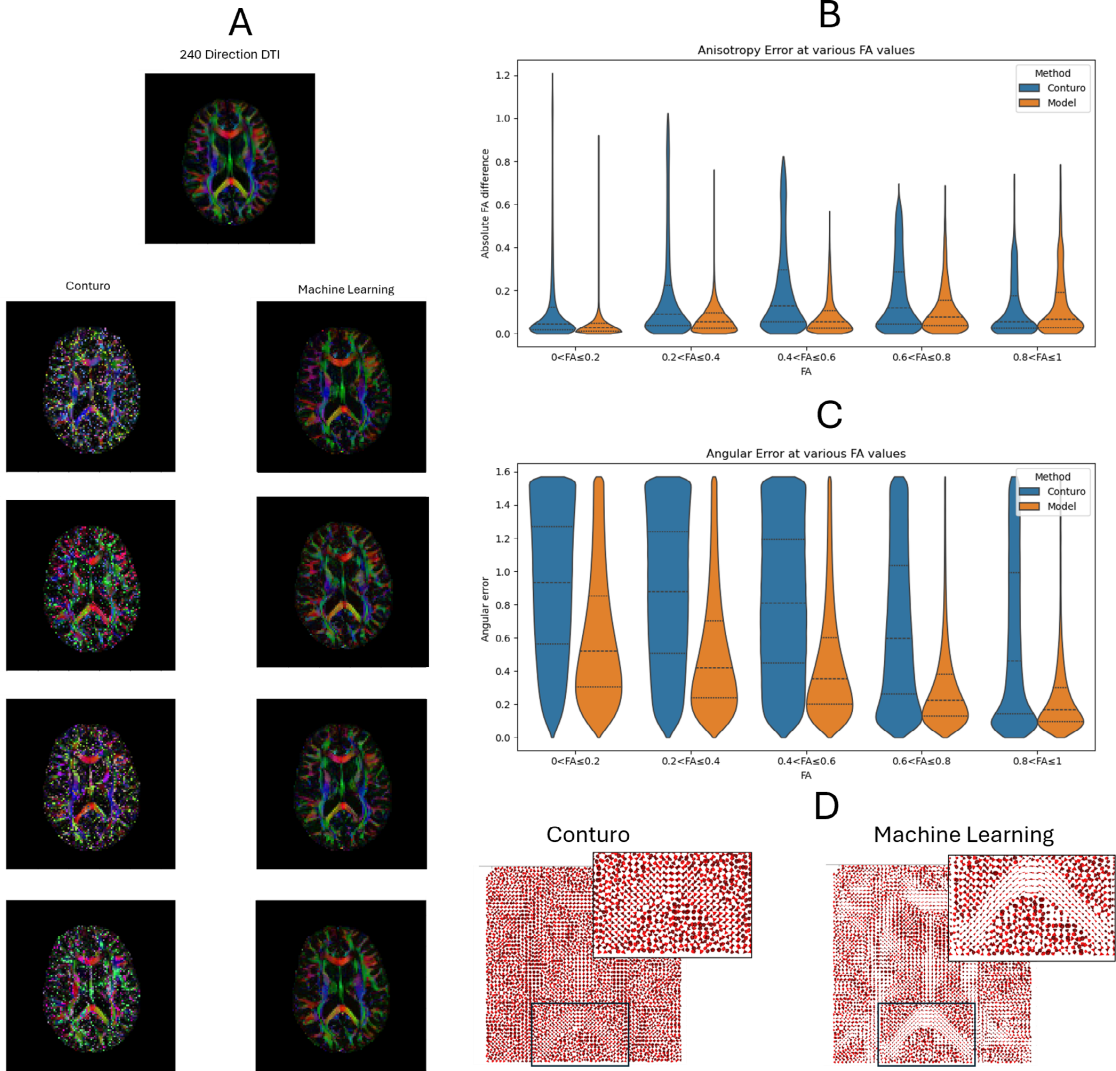
Comparison of the results of the DL model with those of the Conturo et al. method and six direction ordinary least squares on the 240 direction 3T images. (A) The FA‐weighted DTI color maps for various sets of four directions that were fitted with the model and the Conturo et al. method. In addition, it shows various six direction DTI estimates as well. (B) A violin plot of the absolute error in fractional anisotropy for various FA values (the ordinary least squares fit on the 240 directions was used as the ground truth). (C) A violin plot of the angular error at various anisotropy values (while accounting for cylindrical symmetry). (D) The voxelwise cone of uncertainty for each method in the in vivo data [27].

From Figure 10, it can be seen that the proposed model yields lower errors than the standard tetrahedral approach in most scenarios, for both orientation and anisotropy estimates. In particular, the distribution of errors produced by the tetrahedral approach exhibits substantially heavier long tails, indicating a higher prevalence of extreme estimation errors. By contrast, the proposed model produces a more tightly concentrated error distribution, reflecting reduced uncertainty and improved robustness. This reduction in uncertainty is particularly pronounced for orientation estimates in white matter fiber populations. The orientation uncertainty maps show that the proposed method yields significantly lower uncertainty in orientation estimates, particularly in white matter fibers.

## 5 Discussion

The noise susceptibility tests comparing six‐direction linear least squares estimation and the standard tetrahedral approach reveal significant accuracy loss in diffusivity estimates at lower SNR values, especially when using four directions. This renders the standard tetrahedral approach unreliable for anisotropy estimation in LF/ULF scenarios with low SNR. However, the DL model ameliorates the impact of SNR on four‐direction diffusivity estimates. Although deviations from the ground truth persist, particularly at SNR values below 20, the DL results are more coherent than those from the standard tetrahedral approach.

Figures 3 and 4 illustrate errors in the estimation of parallel and radial diffusivities and principal eigenvector orientation when tensors are aligned close to the scanner's axes. While the errors are not eliminated entirely, they are significantly reduced. These tests used non‐cylindrically symmetrical tensors, further highlighting the DL model's superior performance over the standard tetrahedral approach in such cases. These results are supported by the 3T results in Figure 8, where we see variations in the results for different orientations of the tetrahedral vectors, yet the model consistently produces better results than the standard tetrahedral approach.

Importantly, Figure 10 shows that the differences between the two approaches extend beyond mean errors. The standard tetrahedral method exhibits pronounced long‐tailed error distributions for both FA and orientation, reflecting a higher likelihood of extreme failures, particularly in low‐SNR, low‐direction settings where such outliers can dominate downstream analyses. The large FA error ranges observed at low anisotropy in Figure 10C, including values exceeding 1, stem from the same underlying issue. As shown in Figure 2, the tetrahedral fit frequently yields negative eigenvalues at low SNR. While FA is bounded between 0 and 1 for a positive‐definite tensor, negative eigenvalues raise the algebraic upper limit to √(3/2)≈1.225. Consequently, the largest absolute FA errors occur where the “silver‐standard” FA from the 240‐direction dataset is smallest, explaining the broadest error distributions in the 0 < FA < 0.2 and 0.2 < FA < 0.4 bins.

By contrast, the proposed DL‐based approach produces more tightly concentrated error distributions, reflecting improved robustness and a reduced sensitivity to noise, orientation effects, and deviations from model assumptions. This suppression of extreme errors is especially evident in white matter fiber populations, where reliable orientation estimates are critical. We note that Armitage [9] has previously highlighted that for non‐axisymmetric diffusion, the standard tetrahedral approach gives erroneous results, while Lazar et al. [2] highlight the impact of noise and partial volume artifacts on the assumptions of cylindrical symmetry. Despite imposing an assumption of cylindrical symmetry for tensor estimation, our method makes reliable predictions even when the measurements deviate from that assumption.

The digital phantom results in Figure 6 and 64 mT results in Figure 7 highlight the improvement in anisotropy and fiber orientation estimates. In the 64 mT data, there is almost no discernible structure visible with the standard tetrahedral approach, whereas our method reveals tracts even in low SNR cases. It is important to note that these results should not be judged against the visual standards of 3T imaging. Doing so sets an unrealistic benchmark. Rather, they should be viewed in the proper context: a proof‐of‐concept demonstration that meaningful diffusion tensor information could be extracted from low/ultra‐low‐field MRI systems with drastically reduced acquisition times.

As with the broader trajectory of MRI technology, image quality at low field is not static. The field strength may be more or less fixed, but hardware performance is not. In fact, since the time of data acquisition for this study, the vendor of the portable 64 mT system has already iterated twice on its design and construction, improving magnet design, gradient amplitude uniformity, and SNR performance. History has shown us that MRI image quality consistently improves as engineering innovations advance. LF/ULF systems are no exception.

What we present here is not an endpoint, but a starting point. These results highlight the transformative potential of low‐field DT‐MRI for global health, especially in settings where conventional high‐field systems are impractical or unaffordable. With further improvements in hardware and acquisition strategies, this approach could play a major role in the democratization of access to advanced neuroimaging.

We also speculate that the ability to obtain results like those shown in Figure 8, at high field and with minimal acquisition could open the door to novel applications in monitoring dynamic changes in tissue microstructure related to functional activation. Building on recent studies that have demonstrated functional changes in mean diffusivity or ADC [28], our approach offers the possibility of tracking changes in fractional anisotropy (FA) with relatively high temporal resolution, without the need for extensive averaging or lengthy acquisitions. This could enable new research avenues into temporally resolved microstructural dynamics in response to internal or external perturbations, expanding on the early work of [29] on functional diffusion tensor MRI (fDTI). In this context, our method may support continuous or repeated FA monitoring during task engagement or physiological modulation, potentially offering a *microstructural* complement to conventional fMRI.

In cases where there is perfect alignment between the tensors and the axes, there is insufficient information to make accurate predictions, as shown in Figures 3 and 4. However, since the ADCs of all four input signals will be the same (Equations (2a), (2b), (2c), (2d)), the inputs to the model are fixed and predictable, making the outputs consistent and reproducible. This makes it easier to identify ill‐constrained voxels. While errors occurring when the tensor aligns with the scanner's axes are reduced, they are not entirely removed, making our method unsuitable for advanced applications like whole‐brain tractography. Future work could explore Bayesian approaches for uncertainty estimation or incorporating spatial priors to further reduce errors.

## 6 Conclusion

Our research advances the approach of performing DT‐MRI using only four directions. Our refined approach overcomes the numerical instabilities that have hindered the robust application of earlier methods, without introducing significant computational overhead or increased noise sensitivity. The implications of this advancement are particularly noteworthy, suggesting a pathway to significantly reduce DT‐MRI scan durations, being especially valuable in low‐ and ultra‐low‐field MRI applications, where time efficiency and noise management are critical concerns.

## Funding

This work was supported by Wellcome Trust (Wellcome Investigator Award: 96646/Z/11/Z, Wellcome Strategic Award: 104943/Z/14/Z) and the Bill and Melinda Gates Foundation (INV‐047888 (PI Steven Williams)).

## Conflicts of Interest

The authors declare no conflicts of interest.

## Supporting information


**Figure S1:** A graphical representation of the structure used for the two DL models. Each model is a multi‐layer perceptron with four hidden layers between the input and output layers. A rectified linear unit (Relu) is used as the activation function for all the hidden layers.

## Data Availability

The code for the data generation and training of the model, as well as pretrained models can be found at https://github.com/jmametepe/tetrahedral_DTI.
