# Supplementary material for: Four Directions, One Solution: Enabling Rapid Diffusion Tensor MRI for Ultra‐Low Field Using Deep Learning
Source: Magn Reson Med. 2026 May 10;96(3):1413–26. doi: 10.1002/mrm.70417 (PMC13327498; doi:10.1002/mrm.70417)
Supplement: Supplementary file 1 — Figure S1: A graphical representation of the structure used for the two DL models. Each model is a multi‐layer perceptron with four hidden layers between the input and output layers. A rectified linear unit (Relu) is used as the activation function for all the hidden layers. [file MRM-96-1413-s001.docx]

**SUPPORTING INFORMATION**


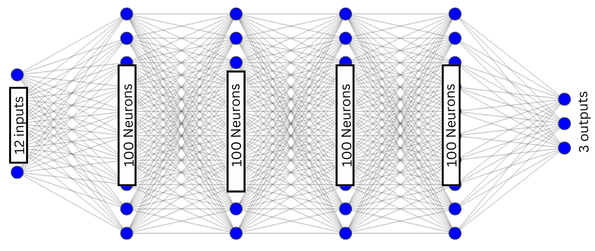


*Supporting Information Figure S1: A graphical representation of the structure used for the two DL models. Each model is a multi-layer perceptron with four hidden layers between the input and output layers. A rectified linear unit (Relu) is used as the activation function for all the hidden layers.*
